# Bearing the mark of pain: mystery in medicine

**DOI:** 10.1186/s13010-023-00134-8

**Published:** 2023-05-19

**Authors:** Karel-Bart Celie, John J. Paris

**Affiliations:** 1 Division of Plastic and Reconstructive Surgery, Department of Surgery, Keck School of Medicine, University of Southern California, Los Angeles, 1510 San Pablo Street, Suite 415, Los Angeles, CA 90033 USA; 2 Uehiro Center for Practical Ethics, University of Oxford, Oxford, UK; 3 Operation Smile Incorporated, Virginia Beach, VA USA; 4 Theology Department, Boston College, 140 Commonwealth Avenue, Chestnut Hill, Boston, MA 02467 USA

**Keywords:** Philosophy, Medical ethics, Suffering, Mystery, Healthcare

## Abstract

Dostoevsky wrote that love in action is a harsh and terrible thing compared to love in dreams. That reality is particularly evident in medicine, where there is an almost universal, involuntary participation of physicians and other healthcare workers in the suffering of their patients. This paper explores this phenomenon through the paradigm of ‘mystery’ as explained by the French existentialist philosopher Gabriel Marcel. A mystery is different from a problem in the sense that the former requires the active immersion of the person involved in order to be truly experienced. It is a ‘meta-problem’ that cannot be analyzed objectively and separately from the person that it affects, without changing the nature of the thing experienced. The authors contend that the human suffering encountered in medicine is one such phenomenon, and the paper draws on illustrations of this concept in art and literature. Awareness of the subtle but important difference between mystery and problem may help physicians better understand their personal entanglement with the suffering of patients.

## Main text

Dostoevsky described love in action as a harsh and dreadful thing compared to love in dreams [1]. The phrase appears superficially contradictory; it is truth “standing on its head to gain attention” in the manner that G.K. Chesterton described paradox [2]. By calling our attention in this way, Dostoevsky highlights that putting love into action means committing oneself to the plight of those who suffer. To serve those who suffer is to *participate *in their suffering, and in few fields is this participation as evident as in medicine. The recent global pandemic has highlighted the widespread burden of suffering and its impact on the burnout of healthcare providers the world over [3, 4], even in regions that have emerged from a lock-down state [5].

The fact is that the human affliction which the medical profession has as its mission is a particular type of problem. It may be what some existentialists have labeled a ‘mystery’: a phenomenon that defies objective analysis.

The distinction between a ‘problem’ and a ‘mystery’ was most clearly stated by the French philosopher Gabriel Marcel. A mystery is an element that ceases to be what it is as soon as attempts are made to separate the *thing* from *its experience*. It is a meta-problem, one that “encroaches on its own data” [6]. An example Marcel emphasizes is evil. He writes, “Evil which is stated or observed is no longer evil which is suffered: in fact, it ceases to be evil. In reality, I can only grasp it as evil in the measure in which it *touches* me—that is to say, the measure in which I am involved” [6]. In contrast to this characterization of mystery, Marcel described a ‘problem’ as something that does *not* require involvement from the observer. One is able to analyze a problem the way one might study a mathematical equation, or some other puzzle, quite independently of any necessary experiential involvement. The observer of a problem is not bound to the experience of the problem. They are able to remain outside of it, before it, beyond it. On the other hand, a person cannot separate themselves from a mystery in order to examine it without changing the nature of the thing being examined. A mystery is similar to something in the peripheral vision that disappears as soon as it is looked at directly. For example, an explanation of love paints a picture that merely resembles the active experience of love, just as a book on laughter can be entirely devoid of humor. Sartre alluded to something similar when describing self-knowledge, or the Ego: “It is never seen except ‘out of the corner of one’s eye’. The moment I turn my gaze on it... it vanishes” [7]. People, like the mysteries described by Marcel, may be knowable to themselves and others only through experiential involvement. 

The distinction has practical implications. Some authors have pointed out the disconnect with reality that occurs when a problem is substituted for a mystery. For example, Albert Camus writes about the glorification of suffering by the village priest—who is frequently referred to as a ‘scholar’—in a town beset by plague. When discussing the priest’s assertions regarding the redemptive qualities of suffering, one of the town doctors responds:

“[The priest] is a man of learning, a scholar. He hasn’t come in contact with death; that’s why he can speak with such assurance of the truth—with a capital T. But every country priest who visits his parishioners and has heard a man gasping for breath on his deathbed thinks as I do. He’d try to relieve human suffering before trying to point out its excellence” [8].

The country priest who visits his parishioners in times of plague comes face to face with the horror of suffering by way of his experience with it; it remains a mystery to him. The scholar’s notion of suffering, in contrast, is a problem that remains objective and abstract.

Camus’ literary example suggests that the human suffering which lies at the core of medicine falls within the category of mystery. Dealing with it necessarily requires experiential involvement beyond what would be required of an ordinary problem. Indeed, the central theme of medicine is not the diagnostic puzzle presented by each patient, essential as such puzzle-solving skills may be to a good physician. Physicians are not problem-solvers *only*. At the core of the art of medicine lies a commitment to humankind and its brokenness; a devotion to caring for people in the many intangible ways they experience suffering. This transforms the character of the profession from an occupation to a calling.

**Fig. 1:**
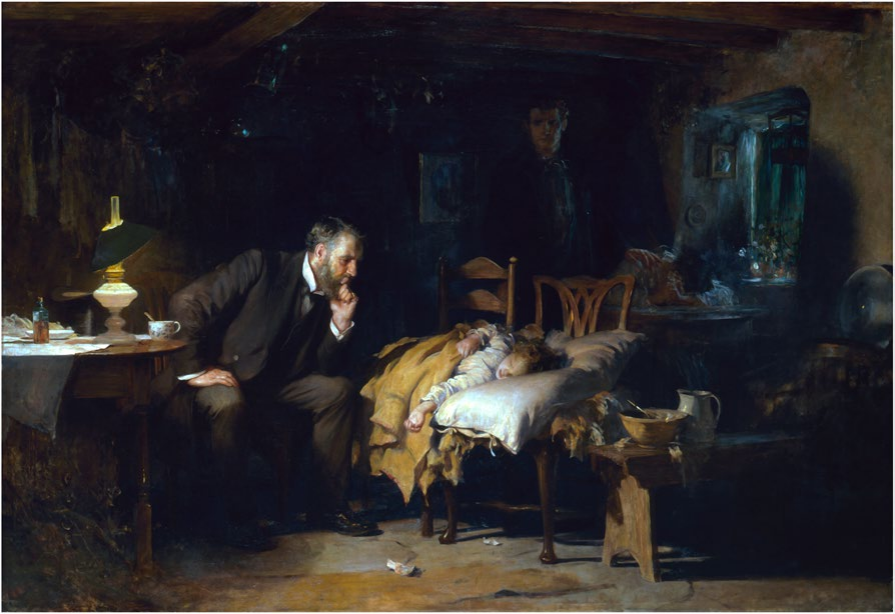
Sir Luke Fildes, The Doctor, 1891. Tate Gallery, London, United Kingdom. (This image was purchased from the Tate Gallery and licensed for academic print distribution.)

**Fig. 2:**
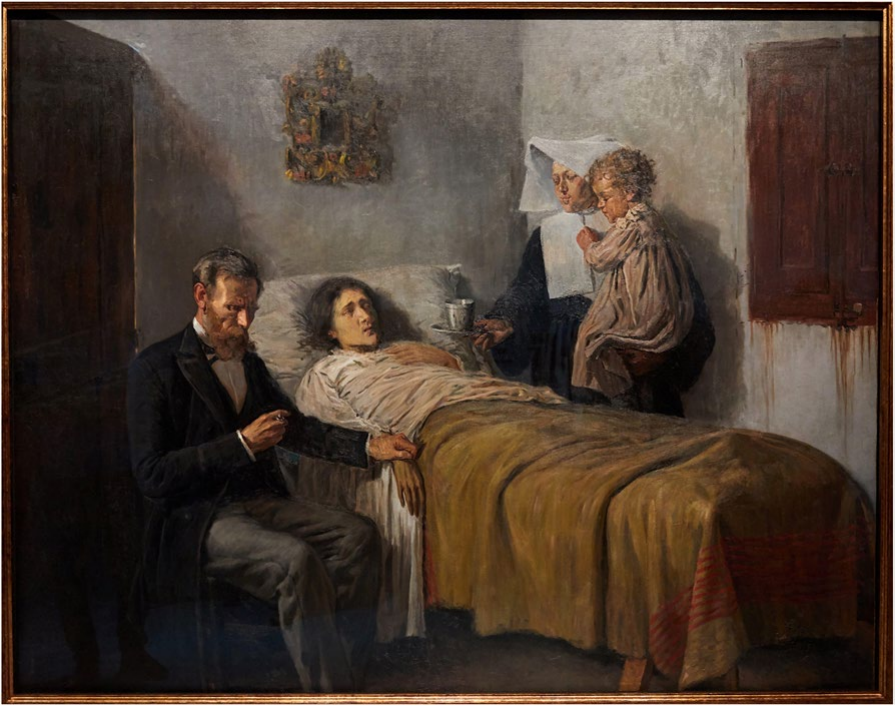
Pablo Picasso, Science and Charity, 1897. Museu Picasso, Barcelona, Catalonia, Spain. (This image was purchased from Alamy Inc. and licensed for academic print distribution.)

The Hippocratic corpus also alludes to this, as indicated by the statement: “Wherever the art of medicine is loved, there is also a love of humanity” [9]. Once it is understood that a love of humanity is the foundation of medicine’s mission to alleviate suffering, there is no way to approach the question of suffering without personal engagement. This is why the bond between patient and physician is held in such high regard. The regard is not due to a contractual relationship, rather, it is because the relationship is a quintessential symbol of the love of one’s neighbor and the personal engagement that this entails. This love, translated to *suffering-with* the patient and family, has been captured particularly well by some artists. Sir Luke Fildes portrayed a doctor in a dimly lit room, chin in hand, hunched over the sickly shape of a little girl on a makeshift bed made of two chairs in his 1890 painting titled ‘The Doctor’ (Fig. 1). The same sentiment was evoked by 15-year-old Pablo Picasso in his painting ‘Science and Charity,’ which depicts a sick woman in bed attended on the right by her physician studiously taking her pulse, and to the left by a nun holding the woman’s child and handing her tea (Fig. 2). To lose sight of the personal engagement portrayed in these paintings would be to see the physician as a highly skilled technician who extirpates diseases, not someone who treats people. That would be problematic not only for its narrow focus, since the mere absence of disease does not imply good health [10], but also because it disregards the patient. We do not eradicate pain at the expense of the pained, or battle disease with no regard to side effects, for the same reason. Caring for patients demands a *suffering-with*, rather than simply the elimination of pain and disease.

The act of suffering-with has best been described by those who have witnessed great hardship. During his account of disease and the regional wars that were then ravaging what is now Gabon, Albert Schweitzer somberly observed that to be human was to be “subject to that terrible lord whose name is Pain” [11]. He elaborated that: “Those who have learnt by experience what physical pain and bodily anguish mean, belong together all the world over… One and all they know the horrors of suffering to which man can be exposed, and one and all they know the longing to be free from pain” [11]. The people united by this bond he labeled as members of the “Fellowship of those who bear the Mark of Pain” [11]. Anyone who has escaped death or anguish with the help of medical care, as well as their family members, belong to this Fellowship. Due to the experiential involvement of mystery, the concept of this Fellowship could be said to extend to the health workers themselves. To care for the suffering is to *suffer-with*; to be branded by pain due to the entanglement that mystery requires. The resulting ‘mark of pain’ unites practitioners of the art of medicine the world over, and it also initiates them into a common Fellowship.

It is worth noting that mysteries are lived experiences, and that they are therefore known only in an *active* way. They are *beholden* more than they are *known*. In an age in which knowledge has revolutionized our way of life, it is occasionally necessary to explicitly assert the primacy of lived experience over impersonal reason when it occurs. Dostoevsky is well known for highlight the importance of orthopraxy before orthodoxy. The realm of mystery is one such instance. In a metaphysical way, the doing of the right thing precedes an understanding of what ‘right’ means. It is understood *through *experience: it could be said that the experience of mystery informs rational thought in an intuitive way. This has been suggested by thinkers throughout the ages, such as when Aristotle asserted that “the end aimed at” in ethics is not knowledge, but action [12].

The fundamental connection between mystery and action may also be why we say the art of medicine is *practiced*. It is more profoundly practiced in the encounter with a patient than in the act of administering a medication, providing a surgical procedure, or having a diagnostic epiphany. These latter activities are merely instrumental means to a more important end, which is the care of the patient—the love of one’s neighbor put into action in a concrete way. Hence physicians throughout the ages have been able to labor for the benefit of their patients, despite deficiencies in their instruments (e.g., laboratory tests, medications) and scientific knowledge. Certainly, the knowledge brought to light by the scientific revolution has dramatically improved the *equipment* that is available to physicians in their quest to cure, to heal, and to care. The point, however, is that their quest to care for persons is what drove the need for the tools in the first place. Tools address a problem. Physicians address the suffering of people, which is a mystery.

As Roger Scruton put it, “The history of philosophy abounds in thinkers, who, having concluded that the truth is ineffable, have gone on to write page upon page about it” [13]. This has been an attempt to express the ‘ineffable’. It is a testament to the presence of mystery that the existentialists have been better at *showing* us what other philosophers for centuries have been *telling* us. Novels such as *The Brothers Karamazov* or *The Plague* are such potent communicators precisely because they vicariously immerse the reader into the experience of mystery. We are *brought* to the parishioners besotted with plague, and not just told about them from the pulpit.

Sir William Osler wrote that: “The good physician treats the disease; the great physician treats the patient who has the disease” [14]. Understood in terms of the concept of ‘mystery’, the great physician is characterized by entanglement with the patient and their suffering. Dostoevsky’s words ring true for this reason. Love in action is harsh because it requires an active giving of oneself. It is dreadful because it demands a willingness to endure suffering alongside the afflicted. To care for patients is to be branded by their afflictions, and to thereby become members of the Fellowship of those who bear the mark of pain.

## Data Availability

Not applicable.
